# Melanogenic Effects of Maclurin Are Mediated through the Activation of cAMP/PKA/CREB and p38 MAPK/CREB Signaling Pathways

**DOI:** 10.1155/2019/9827519

**Published:** 2019-12-22

**Authors:** Young Sun Hwang, Sae Woong Oh, See-Hyoung Park, Jienny Lee, Ju Ah. Yoo, Kitae Kwon, Se Jung Park, Jangsoon Kim, Eunbi Yu, Jae Youl Cho, Jongsung Lee

**Affiliations:** ^1^Department of Dental Hygiene, College of Health Science, Eulji University, Seongnam City, 13135 Gyunggi Do, Republic of Korea; ^2^Molecular Dermatology Laboratory, Department of Integrative Biotechnology & Biocosmetics Research Center, College of Biotechnology and Bioengineering, Sungkyunkwan University, Suwon City, 16419 Gyunggi Do, Republic of Korea; ^3^Department of Bio and Chemical Engineering, Hongik University, 30016 Sejong City, Republic of Korea; ^4^Viral Disease Research Division, Animal and Plant Quarantine Agency, 177 Hyeoksin 8-ro, Gimcheon City, 39660 Gyeongsangbuk Do, Republic of Korea; ^5^Molecular Immunology Laboratory, Department of Integrative Biotechnology, College of Biotechnology and Bioengineering, Sungkyunkwan University, Suwon City, 16419 Gyunggi Do, Republic of Korea

## Abstract

Melanogenesis is the biological process which the skin pigment melanin is synthesized to protect the skin against ultraviolet irradiation and other external stresses. Abnormal biology of melanocytes is closely associated with depigmented skin disorders such as vitiligo. In this study, we examined the effects of maclurin on melanogenesis and cytoprotection. Maclurin enhanced cellular tyrosinase activity as well as cellular melanin levels. We found that maclurin treatment increased the expression of microphthalmia-associated transcription factor (MITF), tyrosinase-related protein- (TRP-) 1, TRP-2, and tyrosinase. Mechanistically, maclurin promoted melanogenesis through cyclic adenosine monophosphate (cAMP) response element binding (CREB) protein-dependent upregulation of MITF. CREB activation was found to be mediated by p38 mitogen-activated protein kinase (MAPK) or cAMP-protein kinase A (PKA) signaling. In addition, maclurin-induced CREB phosphorylation was mediated through the activation of both the cAMP/PKA and the p38 MAPK signaling pathways. Maclurin-induced suppression of p44/42 MAPK activation also contributed to its melanogenic activity. Furthermore, maclurin showed protective effects against H_2_O_2_ treatment and UVB irradiation in human melanocytes. These findings indicate that the melanogenic effects of maclurin depend on increased MITF gene expression, which is mediated by the activation of both p38 MAPK/CREB and cAMP/PKA/CREB signaling. Our results thus suggest that maclurin could be useful as a protective agent against hypopigmented skin disorders.

## 1. Introduction

In recent years, an increase in fine dust caused by industrialization, abnormal climate change, and ozone layer destruction has created conditions that can cause damage to the human body, especially the skin. To reduce the damage caused by external stress factors, the skin biosynthesizes melanin, a skin pigment. This process of skin pigment synthesis is called melanogenesis. However, various stresses can cause defects in melanogenesis, leading to depigmentation skin disorders such as vitiligo [1]. Depigmented skin disorders have been widely studied, but their mechanisms remain largely unknown.

Melanogenesis plays a critical protective role against photocarcinogenesis in the skin [2]. Skin pigmentation depends on several factors, including the type, production, and distribution of melanin, the melanocyte number, the enzymatic activity of melanogenic proteins [3], melanocyte dendricity [4], and melanosome transfer [5]. Tyrosinase-related protein- (TRP-) 1, TRP-2, and tyrosinase are melanocyte-specific enzymes involved in melanin biosynthesis. The expression of those melanogenic genes is regulated by microphthalmia-associated transcription factor (MITF), which has a basic helix-loop-helix leucine zipper [6]. Specifically, MITF increases the expression of TRP-1, TRP-2, and tyrosinase by binding to the M-box that the three genes share in their promoter regions.

Various stimuli are involved in the induction of pigmentation. They include ultraviolet irradiation, abnormal release of *α*-melanocyte-stimulating hormone, inflammation, and rubbing of the skin [7]. In addition, skin melanogenesis is mediated via several melanogenic signaling pathways, including p38 mitogen-activated protein kinase (MAPK) signaling, the cyclic adenosine monophosphate- (cAMP-) mediated pathway, the protein kinase C- (PKC-) mediated pathway, the phosphatidylinositol 3 kinase (PI3K)/AKT signaling, and the p44/42 MAPK pathway. That is, p38 MAPK phosphorylation increases the expression of MITF and tyrosinase, leading to the induction of melanin synthesis [8]. Elevated levels of intracellular cAMP also result in the activation of protein kinase A (PKA), which phosphorylates the cAMP-responsive element binding (CREB) binding protein and CREB protein, increasing the expression of MITF [9]. The contribution of the protein kinase C- (PKC-) mediated pathway to melanogenesis is unclear; the phosphatidylinositol 3 kinase (PI3K)/AKT signaling pathway suppresses melanogenesis by reducing the expression of tyrosinase, MITF, and TRPs [10, 11]. p44/42 MAPK reduces melanin synthesis by degrading the tyrosinase protein [12].

**Figure 1:**
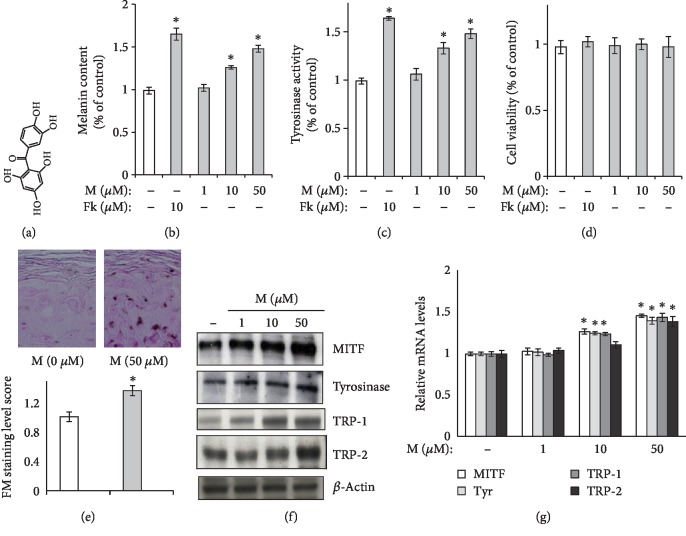
Melanogenesis was upregulated in human epidermal melanocytes. (a) Chemical structure of maclurin. (b, c) Maclurin increased both the (b) melanin level and (c) activity of cellular tyrosinase. ^∗^*p* < 0.05 vs. control group. (d) Maclurin did not show cytotoxicity at the concentrations tested. (e) Maclurin increased melanin levels in the reconstructed epidermis. (f) Maclurin increased the protein levels of melanogenesis-related genes: MITF, TRP-1, tyrosinase, and TRP-2. (g) Maclurin increased the mRNA levels of melanogenesis-related genes: TRP-1, TRP-2, MITF, and tyrosinase. ^∗^*p* < 0.05 vs. control group. M: maclurin; Fk: forskolin.

The structural name of maclurin is (3,4-dihydroxyphenyl)-(2,4,6-trihydroxyphenyl) methanone, and it is a member of the benzophenone family (Figure 1(a)). It exists in *Morus alba* (white mulberry) and *Garcinia mangostana* (purple mangosteen). Although previous reports demonstrated that maclurin has antioxidant and antimetastatic effects, inhibiting cancer cell migration and invasion in non-small-cell lung cancer cells [13–15], the involvement of maclurin in skin cell biology has not been elucidated. Specifically, its effects on the signal transduction pathways of melanogenesis in human epidermal melanocytes have not been previously reported.

In the present study, we investigated the effects of maclurin on melanogenesis and its action mechanism in human epidermal melanocytes.

## 2. Materials and Methods

### 2.1. Materials and Cell Viability Assay

Moloney murine leukemia virus reverse transcriptase, random primers, and TRIzol reagent were purchased from Invitrogen (Carlsbad, CA, USA). TaqMan reverse transcription polymerase chain reaction (RT-PCR) reagents, primers, and probes were obtained from Applied Biosystems. Phorbol myristate acetate, anti-*β*-actin, forskolin, SB202190, KT5720, and antityrosinase were obtained from Sigma-Aldrich (St. Louis, MO, USA). Anti-TRP-1, anti-MITF, and anti-TRP-2 were obtained from NeoMarkers (Fremont, CA, USA). Maclurin (purity: 99%) was purchased from Chirochem (Daejeon, Korea). Human epidermal melanocytes were purchased from Cascade Biologics (Portland, OR, USA) and cultured in Medium 254 (Cascade Biologics) supplemented with human melanocyte growth supplement at 37°C and 5% CO_2_. For the measurement of cell viability, we used the 5-bromo-2′-deoxyuridine (BrdU) incorporation assay [16]. BrdU incorporation was detected by an enzyme-linked immunosorbent assay (ELISA) using a BrdU Cell Proliferation Assay Kit (Cell Signaling Technology, Danvers, MA, USA) according to the manufacturer's instructions.

### 2.2. Assay for Melanin Content

Melanin content was determined as previously described by Lee et al. [17]. Briefly, human epidermal melanocytes were treated with maclurin and then washed with PBS, harvested, and subjected to a melanin content assay. The harvested cell pellets were dissolved in 1 N NaOH (60°C) for 1 h, and the colorimetric analysis was conducted at 475 nm using a spectrophotometer. Melanin content is presented as the ratio of the maclurin-treated group to the control group (% of control).

### 2.3. Cellular Tyrosinase Activity Assay

The enzymatic activity of cellular tyrosinase was measured as described previously [18], with slight modifications. Briefly, human melanocytes were treated with maclurin and then washed with PBS and homogenized with buffer solution (50 mM sodium phosphate (pH 6.8), 1 mM phenylmethanesulfonyl fluoride, and 1% Triton X-100) at 4°C in a Dounce homogenizer. To collect the supernatant as the source of crude cellular tyrosinase, the lysates were centrifuged at 15,000 rpm for 15 min. A Bradford assay was used to measure the protein content in the supernatant, and bovine serum albumin was used as the protein standard. Cellular tyrosinase activity was then determined by measuring the absorbance at 470 nm. In this assay, 3,4-dihydroxyphenylalanine was used as a tyrosinase substrate.

### 2.4. Histochemistry of Reconstructed Epidermis

A reconstructed epidermis (MatTek) was incubated in a medium containing 50 *μ*M maclurin for 2 weeks, with the medium replaced every two days. The epidermis was fixed with 4% formalin in PBS and subjected to Fontana-Masson staining to determine the levels of melanin pigments. For staining of the reconstructed epidermis, a Fontana-Masson Staining Kit (American MasterTech, Lodi, CA) was used, and quantification of the Fontana-Masson staining was conducted using ImageJ software (NIH, Bethesda, MD, USA) [19].

### 2.5. Analysis of mRNA Levels Using Real-Time RT-PCR

The real-time RT-PCR analysis was performed using an ABI7900HT Instrument (Applied Biosystems, Waltham, MA, USA). For the TaqMan analysis, predesigned or optimized assays on demand (Applied Biosystems) were used, including MITF (ID: Hs01117294_m1), tyrosinase (ID: Hs00165976_m1), TRP-1 (ID: Hs00167051_m1), TRP-2 (ID: Hs01098278_m1), glyceraldehyde-3-phosphate dehydrogenase (GAPDH) (ID: Hs00266705_g1), hypoxanthine-guanine phosphoribosyltransferase (HPRT) (Hs02800695_m1), and 18S (Hs03003631_g1). The data were analyzed using ABI Sequence Detector Software version 2.0 (Applied Biosystems). Total RNA was extracted from cells using TRI reagent® according to the manufacturer's instructions and stored at -70°C until use. cDNA was synthesized from total RNA (1 *μ*g) using MuLV reverse transcriptase according to the manufacturer's instructions. The real-time RT-PCR analysis was conducted as previously described [20]. The results were normalized to the expression level of endogenous GAPDH and were also tested against two additional housekeeping genes (18S and HPRT). We found that the results were not significantly different from those obtained using GAPDH. Expression levels of the target genes were normalized to the levels observed in the controls. The results were verified by repeating each experiment four times in triplicate.

### 2.6. Western Blot Analysis, PKA Kinase Activity Assay, and Assay for Phosphorylated CREB Protein Levels

Levels of melanogenic proteins were measured by Western blot analysis after treatment with maclurin for 5 days. The Western blot analysis was conducted as previously described [9]. In brief, proteins separated using sodium dodecyl sulfate-polyacrylamide gel electrophoresis were transferred to polyvinylidene difluoride membranes (Bio-Rad, Hercules, CA, USA), which were then probed with appropriate primary and secondary antibodies (phospho CREB, phospho p44/42 MAPK, phospho nuclear factor-kappa B (NF-*κ*B) p65 (Ser536), phospho c-Jun N-terminal kinase (JNK), phospho p38 MAPK, NF-*κ*B p65, tyrosinase, CREB, TRP-2, p38 MAPK, MITF, p44/42 MAPK, TRP-1, and JNK). Finally, protein levels were measured using an enhanced chemiluminescence kit (Amersham, Piscataway, NJ, USA). A PKA kinase activity assay kit (Stressgen, Ann Arbor, MI, USA) was used according to the manufacturer's protocols to measure PKA kinase activity [21]. A PathScan® Phospho-CREB (Ser133) sandwich ELISA kit was used according to the manufacturer's instructions to determine phosphorylated CREB protein levels [22].

### 2.7. UV Irradiation and Measurement of Intracellular Reactive Oxygen Species (ROS) Levels

Before UV irradiation, cultured cells were incubated with 1% serum medium for 12 h. Then, the cells were exposed to UVB radiation at an intensity of 20 mJ/cm^2^ (Luzchem Research Inc., Ottawa, Canada) [23]. After UVB irradiation, the cells were treated with maclurin. After incubation for the indicated time, cells were harvested and subjected to the ROS assay. To exclude the possibility that UVB irradiation was cytotoxic, cell viability was evaluated using the 3-(4,5-dimethylthiazol-2-yl)-2,5-diphenyltetrazolium bromide (MTT) assay. Briefly, cells were treated with MTT (0.1 mg/mL) for 3 h at 37°C in a 5% CO_2_ atmosphere. The medium was then removed, and the cells were solubilized with dimethyl sulfoxide (1 mL). After complete solubilization, the presence of blue formazan was evaluated spectrophotometrically by measuring the absorbance at 570 nm. For ROS measurement, cells were preloaded with 10 *μ*M 2′,7′-dichlorodihydrofluorescein diacetate for 30 min and then irradiated with UVB and treated with maclurin. The cells were lysed using 0.1% Triton X-100 solution, and the fluorescence intensity of the lysate was determined using a fluorometer (INFINITE M200, Tecan, Männedorf, Switzerland). In addition, ROS levels were observed directly using an Evos fluorescent microscope [24].

### 2.8. Statistical Analysis

For statistical analysis of the collected data, one-way analysis of variance was used, and statistical significance was accorded to a *p* value less than 0.05.

## 3. Results

### 3.1. Maclurin Promotes Melanogenesis in Human Epidermal Melanocytes

Maclurin concentration dependently increased both melanin content (Figure 1(b)) and cellular tyrosinase activity (Figure 1(c)) without any cytotoxicity at the concentrations tested (Figure 1(d)). In these experiments, forskolin was introduced as a positive control [17] because it increases both melanin content and cellular tyrosinase activity. In the Fontana-Masson staining and photography analysis, we found that maclurin treatment increased the level of melanin in the reconstructed epidermis (Figure 1(e)). The protein levels of MITF, tyrosinase, TRP-1, and TRP-2 increased with maclurin treatment (Figure 1(f)), and so did their mRNA levels (Figure 1(g)).

### 3.2. Maclurin Activates cAMP/PKA/CREB Signaling

**Figure 2:**
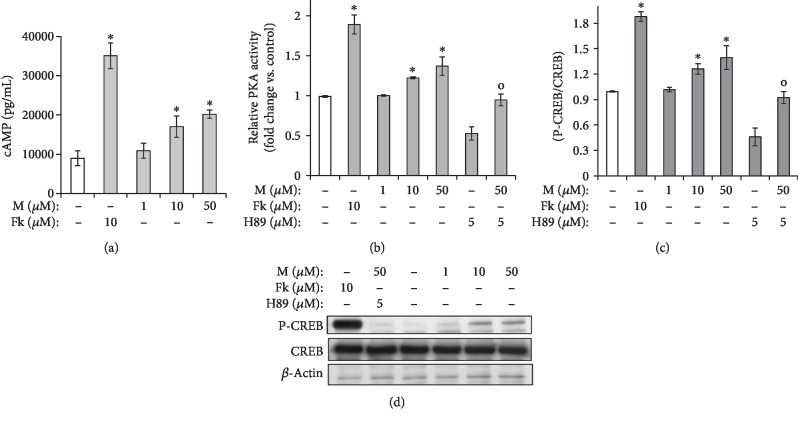
Maclurin activated the cAMP-PKA-CREB pathway. (a) cAMP production assay and (b) ELISA were performed to analyze PKA activity. (c) The CREB phosphorylation levels were measured using an ELISA kit and confirmed by (d) a Western blot analysis. ^∗^*p* < 0.05 vs. control group, ^o^*p* < 0.05 vs. maclurin (50 *μ*M)-treated control. M: maclurin; Fk: forskolin.

The cAMP/PKA/CREB signaling pathway is well characterized in melanogenic signaling [9]. Therefore, we investigated the effect of maclurin on signaling molecules in that pathway using ELISA for cAMP, PKA activity, and phosphorylated CREB and a Western blot analysis to confirm the phosphorylated CREB ELISA results. As shown in Figures 2(a)–2(c), maclurin treatment increased cAMP production, PKA activity, and CREB phosphorylation. The effect of maclurin on CREB phosphorylation was also confirmed in the Western blot analysis (Figure 2(d)). Those molecular effects were attenuated by treatment with H89 (a PKA inhibitor) (Figures 2(b)–2(d)). In these experiments, forskolin and H89 were introduced as a positive control and a negative control, respectively [17]. Forskolin increased cAMP production, PKA activity, and CREB phosphorylation, and H89 reduced PKA activity and CREB phosphorylation.

### 3.3. p38 MAPK and p44/42 MAPK Are Affected by Maclurin

**Figure 3:**
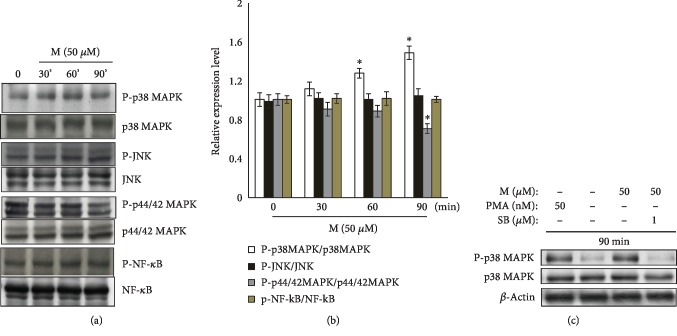
Maclurin activated p38 MAPK and inhibited p44/42 MAPK. (a) Maclurin activated p38 mitogen-activated protein kinase (MAPK) and inhibited p44/42 MAPK but not JNK or NF-*κ*B. ^∗^*p* < 0.05 vs. control group. (b) A Western blot densitometric analysis was performed. (c) SB203580 attenuated the effects of maclurin on p38 MAPK phosphorylation. M: maclurin; PMA: phorbol 12-myristate 13-acetate; SB: SB203580; P-NF-*κ*B: phospho-NF-*κ*B p65 (Ser536); NF-*κ*B: NF-*κ*B p65.

The MAPKs and NF-*κ*B are involved in melanin synthesis, so we examined their involvement in maclurin-induced melanogenesis. Among the MAPKs tested, maclurin had no effect on JNK, but it did affect p38 MAPK and p44/42 MAPK. Specifically, maclurin induced the phosphorylation of p38 MAPK and suppressed the phosphorylation of p44/42 MAPK (Figures 3(a) and 3(b)). The effects of maclurin on p38 MAPK were attenuated by treatment with SB203580 (a p38 MAPK inhibitor) (Figure 3(c)). Maclurin had no effect on NF-*κ*B activation (Figures 3(a) and 3(b)).

### 3.4. The Effects of Maclurin on Pigmentation Are Mediated through the Activation of p38 MAPK and cAMP/PKA Signaling

**Figure 4:**
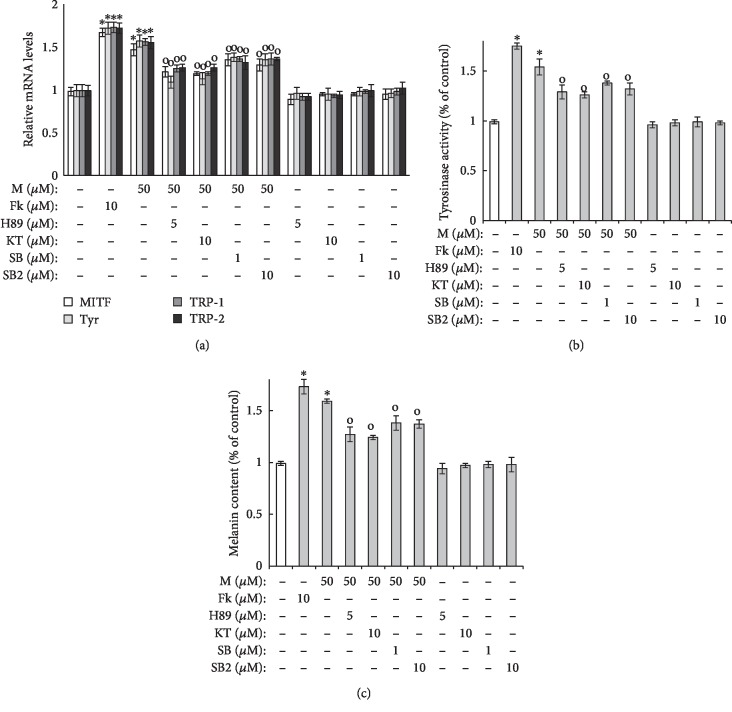
p38 MAPK and cAMP-PKA pathways mediated maclurin-induced melanogenesis. (a) Real-time PCR analysis, (b) cellular tyrosinase activity assay, and (c) melanin content assay results. ^∗^*p* < 0.05 vs. control group, ^o^*p* < 0.05 vs. maclurin (50 *μ*M)-treated control. SB: SB203580; SB2: SB202190; KT: KT5720; M: maclurin; Fk: forskolin.

As shown in Figure 4(a), maclurin treatment enhanced the mRNA levels of TRP-1, TRP-2, MITF, and tyrosinase, and that effect was reduced by the p38 MAPK inhibitors SB203580 and SB202190 and the PKA inhibitors H89 and KT5720. Similar results were found in the cellular tyrosinase activity and melanin content assays using SB203580/SB202190 and H89/KT5720. As shown in Figures 4(b) and 4(c), tyrosinase activity and melanin content were increased by maclurin treatment, and those effects were attenuated by SB203580/SB202190 and H89/KT5720.

### 3.5. p38 MAPK and cAMP/PKA Signaling Reciprocally Communicate through CREB Phosphorylation in Maclurin-Induced Pigmentation

**Figure 5:**
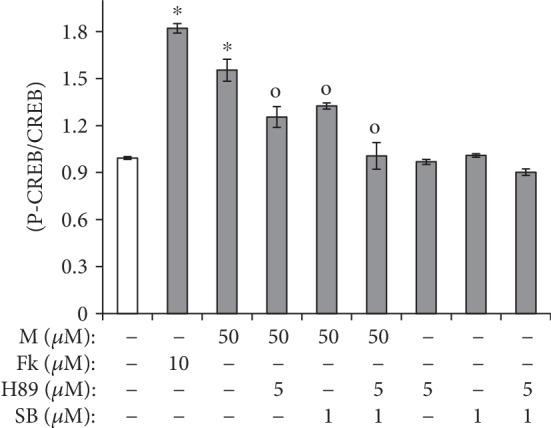
Maclurin activated CREB phosphorylation through both PKA and p38 MAPK activity. Enzyme-linked immunosorbent assays (ELISAs) were performed to analyze protein kinase A (PKA). ^∗^*p* < 0.05 vs. control group, ^o^*p* < 0.05 vs. maclurin (50 *μ*M)-treated control. SB: SB203580; M: maclurin; Fk: forskolin.

To examine the relationship between cAMP/PKA signaling and p38 MAPK signaling in maclurin-induced melanogenesis, we examined the effects of H89 and SB203580 on CREB phosphorylation levels. As shown in Figure 5, H89 and SB203580 both reduced the CREB phosphorylation induced by maclurin. In addition, the combined treatment of SB203580 and H89 synergistically inhibited the effects of maclurin on CREB phosphorylation.

### 3.6. The Melanogenic Effects of Maclurin Depend on Increased MITF Gene Expression

**Figure 6:**
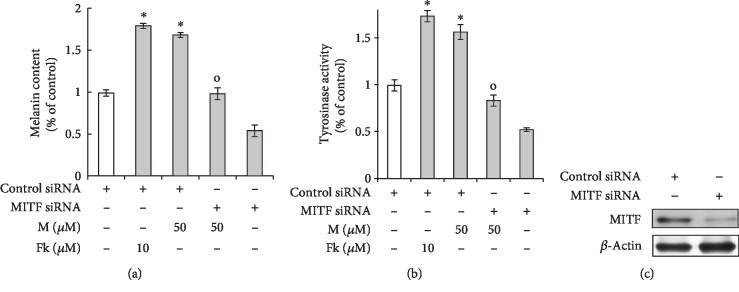
Maclurin-induced pigmentation was attenuated by MITF knockdown. (a, b) Knockdown of MITF attenuated the effects of maclurin on (a) melanin levels and (b) cellular tyrosinase activity. (c) Western blot analysis of MITF. ^∗^*p* < 0.05 vs. control group, ^o^*p* < 0.05 vs. maclurin (50 *μ*M)-treated control. M: maclurin; Fk: forskolin.

We confirmed that the melanogenic effects of maclurin are mediated by upregulation of the MITF gene. As shown in Figures 6(a) and 6(b), we found that knockdown of MITF using siRNA attenuated the effects of maclurin, such as increased melanin levels and tyrosinase activity. MITF siRNA successfully knocked down MITF protein in human melanocytes compared with the level in cells transfected with control siRNA (Figure 6(c)). However, we did not perform related experiments under overexpression of MITF gene. These data indicate that maclurin increases pigmentation by inducing MITF-dependent signaling.

### 3.7. Maclurin Protects Melanocytes against H_2_O_2_ and UVB

**Figure 7:**
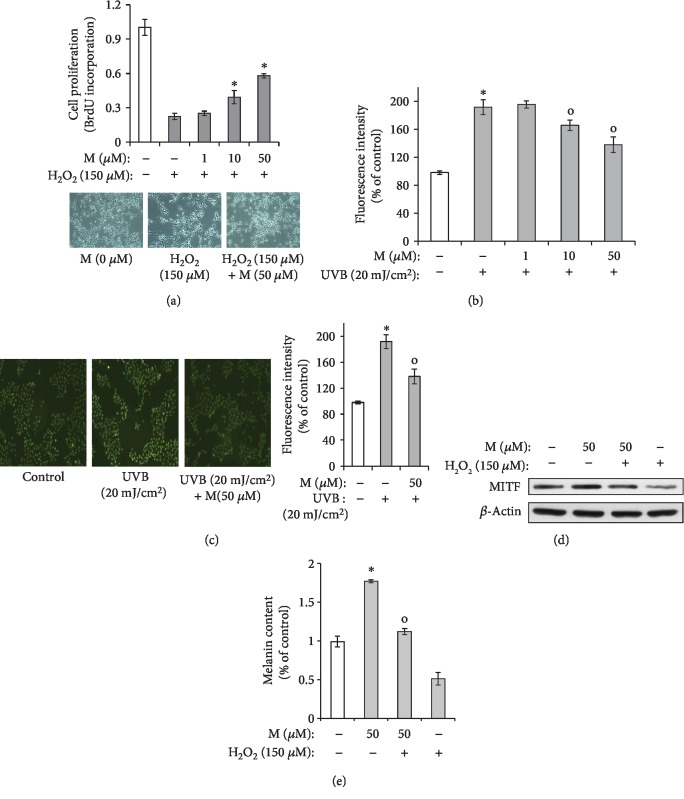
Maclurin protected human epidermal melanocytes against the H_2_O_2_-mediated decrease in cell viability. (a) The BrdU incorporation assay was used to measure the percentage of viable cells 14 h after H_2_O_2_ treatment. ^∗^*p* < 0.05 vs. control group, ^o^*p* < 0.05 vs. H_2_O_2_ (150 *μ*M)-treated control. (b) Effects of maclurin on UVB-induced ROS production in human epidermal melanocytes. ROS-induced dichlorofluorescein (DCF) formation was measured using a spectrophotometer. ^∗^*p* < 0.05 vs. control group, ^o^*p* < 0.05 vs. UVB-irradiated control. (c) ROS-induced DCF fluorescence was visualized by fluorescence microscopy. ^∗^*p* < 0.05 vs. control group, ^o^*p* < 0.05 vs. UVB-irradiated control. (d, e) Effects of maclurin on the H_2_O_2_-induced reduction of MITF protein and melanin content in human epidermal melanocytes. ^∗^*p* < 0.05 vs. control group, ^o^*p* < 0.05 vs. H_2_O_2_-treated control. SB: SB203580; M: maclurin.

As shown in Figure 7(a), we found that maclurin significantly inhibited hydrogen peroxide-induced reductions in cell survival. In addition, UVB-induced production of ROS was attenuated by maclurin treatment (Figures 7(b) and 7(c)). As shown in Figures 7(d) and 7(e), hydrogen peroxide reduced MITF expression levels and melanin content. However, maclurin treatment attenuated the effects of H_2_O_2_. These results indicate that maclurin protects melanocytes from H_2_O_2_-induced damage.

## 4. Discussion

In this study, we have demonstrated the stimulatory effects of maclurin on melanogenesis in human epidermal melanocytes. Maclurin increased MITF gene expression and melanin formation. The results indicate that the melanogenic effects of maclurin occur mainly through MITF upregulation, which is mediated by two different signaling pathways, p38 MAPK and cAMP/PKA signaling. The signals from these two pathways commonly target CREB for phosphorylation, which upregulates MITF. In addition, the results indicate that maclurin has antioxidant and protective effects against H_2_O_2_ and UVB, as well as a pigmenting effect, suggesting that maclurin could be useful as a protective agent against hypopigmented skin disorders.

The MITF protein is essential for the expression of melanogenic genes. In this study, we found that maclurin increased the production of tyrosinase, TRP-1, TRP-2, and MITF. Among them, TRP-1, a 75 kDa protein synthesized in the endoplasmic reticulum, is transported through the Golgi and transferred to melanosomes. The cytoplasmic tail of TRP-1 drives its transfer to the melanosome through a retention signal sequence [25, 26]. TRP-1 is also expressed in the melanosome on the cell surface of melanocytes [27]. In melanogenesis, TRP-1 increases tyrosinase activity by stabilizing it through complex formation. In addition, TRP-1 is involved in the proliferation and morphology of melanocytes [28]. In this study, we found that maclurin upregulated the TRP-1 gene, suggesting that maclurin could contribute to the maintenance of melanocyte biology. TRP-2 (dopachrome tautomerase) converts dopachrome (5,6-dioxo-2,3,5,6-tetrahydro-1H-indole-2-carboxylic acid) to DHICA (5,6-dihydroxyindole-2-carboxylic acid), which affects eumelanin but not pheomelanin synthesis. During pheomelanin synthesis, TRP-2 is reduced [29] and disappears in the bulbar melanocytes of mice producing predominantly pheomelanin [30]. The deletion of the TRP-2 gene in mice leads to the dilution of their coat color and a reduction of the melanin content in their hair shafts [31–34]. Therefore, our results suggest that maclurin contributes to eumelanin synthesis.

The melanogenic signaling pathways include p38 MAPK signaling, the cAMP-mediated pathway, the PKC-mediated pathway, PI3K/AKT signaling, and the p44/42 MAPK pathway. We found that maclurin induced p38 MAPK phosphorylation, and SB203580 attenuated maclurin-induced melanin synthesis. Maclurin also increased the levels of intracellular cAMP, activated PKA, increased the phosphorylation of the CREB protein, and decreased the phosphorylation levels of p44/42 MAPK. However, maclurin showed no effects on the PKC-mediated pathway or the PI3K/AKT signaling pathway. These data indicate that the melanogenic effects of maclurin are mediated by the p38 MAPK, cAMP/PKA, and p44/42 MAPK signaling pathways. Furthermore, we found that maclurin induced CREB phosphorylation through two different signaling pathways, cAMP/PKA and p38 MAPK, suggesting that cAMP/PKA and p38 MAPK-CREB phosphorylation synergistically contribute to maclurin-induced melanogenesis.

Oxidative stress induces depigmentation by downregulating MITF and the MITF-dependent melanogenic enzymes that contribute to melanocyte survival [35]. Vitiligo is a depigmentation skin disorder that results from the death of melanocytes [36]. A combination of antioxidants and melanogenic inducers has shown promising therapeutic efficiency in vitiligo patients, increasing pigmentation levels and cell survival [37]. In our study, maclurin attenuated the reduction in cell survival caused by H_2_O_2_ exposure and reduced UVB-induced ROS production, in addition to its melanogenic effects. Therefore, maclurin could be useful in treating vitiligo.

Although the antimetastatic and antioxidant activities of maclurin have been reported, its activities in skin physiology have not previously been elucidated. In this study, we demonstrated that maclurin has melanogenic activity and that its effects depend on activation of the cAMP/PKA and p38 MAPK signaling pathways. Specifically, maclurin induced melanogenesis by activating CREB through p38 MAPK- and cAMP/PKA-dependent pathways and increasing the expression of the MITF gene.

## 5. Conclusions

Collectively, the results of this study indicate that maclurin regulates melanogenesis by increasing the expression of the MITF gene through the p38 MAPK- and cAMP/PKA-dependent activation of CREB. Therefore, although further research about its efficacy and safety in clinical studies is necessary, maclurin could be used to treat hypopigmented skin disorders such as vitiligo.

## Data Availability

The data used to support the findings of this study are available from the corresponding authors upon request.

## Abbreviations

MITF: Microphthalmia-associated transcription factor
TRP: Tyrosinase-related protein
cAMP: Cyclic adenosine monophosphate
CREB: cAMP-response element binding protein
PKA: Protein kinase A
JNK: c-Jun N-terminal kinase
MAPKs: Mitogen-activated protein kinases
NF-*κ*B: Nuclear factor kappa-light-chain-enhancer of activated B cells
BrdU: 5-Bromo-2′-deoxyuridine
DCF: Dichlorofluorescein.
